# Distinct Proteomic Signatures Driving Progression of Sarcopenia: A Longitudinal Multicohort Study

**DOI:** 10.1002/jcsm.70240

**Published:** 2026-03-04

**Authors:** Sung Hye Kong, Ok Hee Jeon, Ji Yeon Kim, Miji Kim, Jinhee Kim, Seung Shin Park, Hak Chul Chang, Chang Won Won, Dohyun Han

**Affiliations:** ^1^ Department of Internal Medicine Seoul National University Bundang Hospital Seongnam Republic of Korea; ^2^ Department of Internal Medicine Seoul National University College of Medicine Seoul Republic of Korea; ^3^ Department of Biomedical Sciences Korea University College of Medicine Seoul Republic of Korea; ^4^ Department of Health Sciences and Technology, College of Medicine Kyung Hee University Seoul Republic of Korea; ^5^ Department of Preventive Medicine and Public Health Ajou University School of Medicine Suwon Republic of Korea; ^6^ Department of Internal Medicine Seoul National University Hospital Seoul Republic of Korea; ^7^ Department of Family Medicine Kyung Hee University Medical Center Seoul Republic of Korea; ^8^ Elderly Frailty Research Center, Department of Family Medicine, College of Medicine Kyung Hee University Seoul Republic of Korea; ^9^ Department of Transdisciplinary Medicine Seoul National University Hospital Seoul Republic of Korea; ^10^ Department of Medicine Seoul National University College of Medicine Seoul Republic of Korea

**Keywords:** biomarker, complement cascade, lipid metabolism, proteomics, sarcopenia

## Abstract

**Background:**

Sarcopenia is an age‐related condition characterized by progressive muscle mass, strength and physical performance declines, contributing to frailty and adverse health outcomes. Despite increasing interest in molecular biomarkers, longitudinal data with external validation are limited.

**Methods:**

This study applied high‐throughput proteomic analysis to identify and validate biomarkers associated with sarcopenia progression in two independent prospective cohorts. The discovery cohort (*n* = 171) was classified into three groups: (1) nonsarcopenic at both baseline and the 2‐year follow‐up; (2) newly developed sarcopenia; and (3) persistently sarcopenic. The validation cohort (*n* = 93) was followed up for 2 years. Plasma proteomic profiling was conducted using data‐independent acquisition (DIA) mass spectrometry. For the validation cohort, targeted quantification (Hyper Reaction Monitoring‐DIA) and immunoassays were employed to verify key findings. Statistical analyses included multivariable regression and pathway enrichment analysis.

**Results:**

In the discovery cohort, 102 proteins were differentially expressed between groups (*p* < 0.05). Compared to the stable nonsarcopenic group, individuals who developed sarcopenia demonstrated significant APOA1 (fold change −1.42, *p* < 0.001) and KLKB1 downregulation and LECT2 upregulation. Those who remained sarcopenic exhibited persistent B2M (+1.58, *p* < 0.001), S100A9 and LYZ elevation. We identified seven robust protein signatures (LRG1, CST3, TIMP1, C2, ITIH1, AMBP and LYZ) that showed consistent significant associations with sarcopenia components in both cohorts. LRG1 and TIMP1, CST3 and C2 were reproducibly associated with muscle strength, physical performance and muscle mass, respectively. Pathway enrichment analyses consistently highlighted LXR/RXR signalling, acute phase response signalling and complement cascade activation as central mechanisms across these domains.

**Conclusion:**

This study identified and validated plasma protein signatures and pathways associated with sarcopenia progression. Complement activation, acute inflammatory response and lipid dysregulation emerged as central mechanisms. These robustly validated biomarkers may represent targets for early detection and intervention strategies in sarcopenia.

## 1 Introduction

Sarcopenia, which affects up to 50% of adults over 80 years of age, is characterized by an age‐related decline in muscle mass, strength and physical performance [1]. Its pathophysiology is multifactorial, involving chronic inflammation, metabolic dysregulation, neuromuscular degeneration and hormonal alterations [2, 3], which differentially influence muscle mass, strength and physical performance, thereby suggesting that sarcopenia should be assessed as a heterogeneous condition rather than as a singular process [4].

Despite increasing interest in the molecular signatures of sarcopenia, the mechanisms underlying its progression and persistence remain poorly understood. Notably, most studies relied on cross‐sectional designs [5], limiting insights into biomarkers predictive of sarcopenia progression. Therefore, longitudinal studies are essential, as they may enable early diagnosis and targeted interventions to mitigate muscle deterioration [6]. Additionally, the lack of validation in independent cohorts hinders the reproducibility and generalizability of the identified biomarkers [7], challenging the establishment of clinically meaningful proteomic signatures. Robust validation using independent well‐characterized cohorts is necessary to identify biomarkers for sarcopenia diagnosis, prognosis and therapeutic targeting [8]. To address these gaps, our previous work has demonstrated the potential utility of circulating protein biomarkers in characterizing sarcopenia progression [9, 10]. Nevertheless, these studies were limited to targeting known proteins and to single‐cohort designs, highlighting the need for unbiased, proteome‐wide discovery approaches with external validation.

Here, we used high‐throughput data‐independent acquisition mass spectrometry (DIA‐MS) to comprehensively profile plasma proteomes in well‐characterized longitudinal older adult cohorts. Our primary aim was to identify and externally validate the circulating proteins associated with overall sarcopenia progression over time. As a secondary objective, we explored whether distinct protein signatures were associated with declines in specific components of sarcopenia, such as muscle mass, strength and physical performance.

## 2 Methods

### 2.1 Study Participants

**FIGURE 1:**
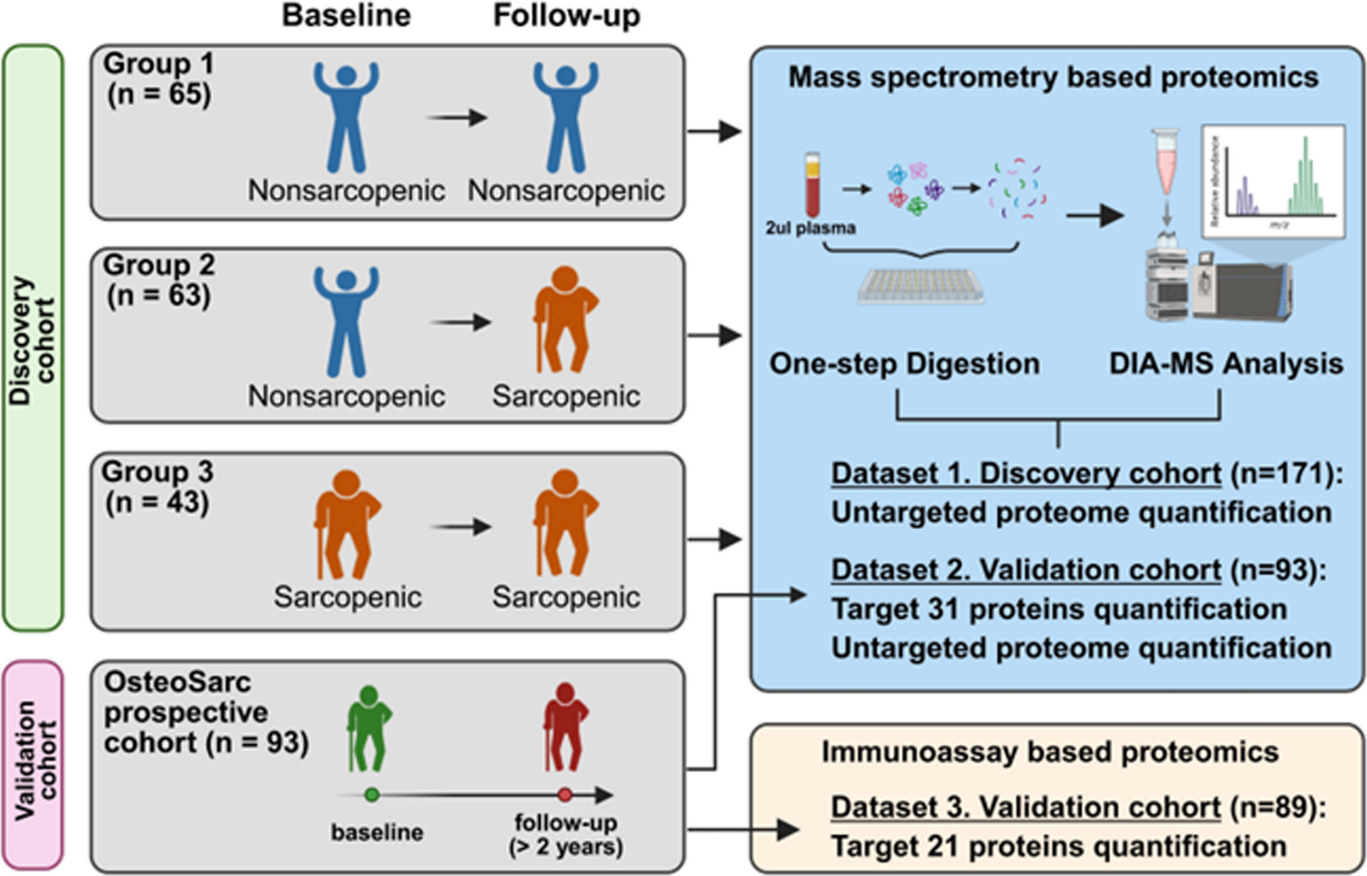
Overview of study participants and analysis.

This study was a longitudinal proteomic investigation consisting of discovery and validation phases (Figure 1). The discovery cohort was derived from the Korean Frailty and Aging Cohort Study (KFACS), a multicentre longitudinal study with baseline assessments conducted between May 2016 and November 2017, including 3014 community‐dwelling older adults aged 70–84 years [11]. Participants were classified into three groups: (1) nonsarcopenic at both baseline and the 2‐year follow‐up (nonsarcopenic, Group 1); (2) newly developed sarcopenia (nonsarcopenic to sarcopenic, Group 2); and (3) persistently sarcopenic (remaining sarcopenic, Group 3). Group 1 comprised 65 age‐ and sex‐matched individuals who remained nonsarcopenic at both assessments and were matched to Group 2. Group 2 included 63 individuals who were nonsarcopenic at baseline but developed sarcopenia during the 2‐year follow‐up period. Group 3 included 43 individuals who were sarcopenic at baseline and remained sarcopenic over the follow‐up period.

Regarding external validation, the Osteoporosis Sarcopenia (OsteoSarc) cohort at Seoul National University Bundang Hospital (SNUBH), Korea, was used [9, 10]. Participants with neuromuscular disorders, including Parkinson's disease, myopathies or muscular dystrophies, were excluded from both cohorts. The OsteoSarc cohort is an ongoing prospective study that gathers baseline and annual assessments of demographic characteristics, bone mineral density, muscle mass, muscle strength and physical performance. Because all participants in this study were independently ambulatory, physical performance was primarily evaluated using the short physical performance battery (SPPB), walking speed and the five‐time sit‐to‐stand test. Individuals with metabolic bone disorders or active malignancies were excluded. The final analysis included 93 participants who were followed up for at least 2 years. This study was approved by the Institutional Review Board of the Seoul National University Bundang Hospital (no. B‐2104‐678‐302), and all participants provided written informed consent, in accordance with the Declaration of Helsinki.

### 2.2 Demographic Variables

Clinical information, including age, sex and comorbidities, such as diabetes, hypertension, cardiovascular disease and cerebrovascular diseases, was collected from hospital records and structured health interviews. The height and body weight of participants dressed in light clothing were recorded to the nearest 0.1 cm and 0.1 kg, respectively. Body mass index (BMI) was calculated as weight divided by height squared (kg/m^2^).

### 2.3 Sarcopenia Assessment

Our study utilized the updated sarcopenia definition provided by the Asian Working Group for Sarcopenia 2019 [1]: low muscle mass (men: < 7.0 kg/m^2^; women: < 5.4 kg/m^2^), low muscle strength (handgrip strength [HGS]; men: < 28 kg; women: < 18 kg) and/or low physical performance. Low physical performance was defined as meeting any of the following criteria: 4‐m usual gait speed < 1.0 m/s, five‐time sit‐to‐stand time ≥ 12 s or SPPB score ≤ 9 [1, 12]. Muscle mass was assessed as appendicular skeletal muscle mass (ASM) measured by dual‐energy X‐ray absorptiometry (Discovery W; Hologic, USA) and calculated as ASM divided by height squared (m^2^). HGS was assessed using a hydraulic hand dynamometer (Grip‐D 5401; Takei Scientific Instruments Co. Ltd., Japan). Each hand was tested three times, with a 60‐s rest between trials, and the highest value was used for analysis. Physical performance was assessed using the SPPB, which comprises three timed components (balance assessment, 4‐m walking speed and the five‐time sit‐to‐stand test), with higher scores indicating better performance. For the balance test, participants maintained side‐by‐side, semi‐tandem and tandem stances for 10 s [13]. Walking speed was measured as usual gait speed (m/s) over a 4‐m course, and the faster of two attempts was used for analysis. In the five‐time sit‐to‐stand test, participants rose from a chair five times consecutively with arms folded across the chest, and total time was recorded. Assistive devices were used if necessary.

### 2.4 Proteomic Analysis

Plasma samples were prepared according to one‐step protein digestion as previously described, with some modifications [14]. The proteomic analysis was conducted in two phases: discovery and validation (Figure 1). For the discovery cohorts, data‐dependent acquisition and DIA were conducted using an Ultimate 3000 UHPLC system (Dionex, Sunnyvale, CA) coupled to a Q‐Exactive Plus mass spectrometer (Thermo Fisher Scientific Inc., Waltham, MA, USA), as previously described, with some modifications [14] (Figure S1).

For the validation cohort, independent datasets were generated using both mass spectrometry and immunoassays (Figure 1). Hyper Reaction Monitoring (HRM)‐DIA analysis was performed using an Orbitrap Exploris 480 coupled with an Ultimate 3000 RSLC system (Dionex, Sunnyvale, CA, USA) consisting of IonOpticAurora LC columns (IonOpticks, Victoria, AUS). From this analysis, we extracted targeted quantitative data using spiked‐in heavy stable‐isotope‐labelled reference peptides for 31 specific proteins, as well as untargeted proteomic profiles. The detailed process is described in the Supporting Information.

Independently, we quantified 21 additional markers using immunoassays to capture well‐established sarcopenia biomarkers and proteins that were challenging to quantify via MS. The panel included apolipoproteins and complement factors: apolipoprotein A‐I (APOA1), apolipoprotein A‐II (APOA2), apolipoprotein B (APOB), apolipoprotein C‐II (APOC2), apolipoprotein E (APOE), complement C3 (C3) and complement C4 (C4). Moreover, we measured well‐known sarcopenia‐related hormones, myokines and inflammatory markers, including growth differentiation factor 15, myostatin, activin A, adiponectin, brain‐derived neurotrophic factor, irisin, tumour necrosis factor alpha, leptin, interleukin‐6 (IL‐6), procollagen type III N‐terminal peptide, dehydroepiandrosterone sulphate, insulin‐like growth factor 1 (IGF‐1), cortisol and high‐sensitivity C‐reactive protein (hs‐CRP). Measurements were performed according to the manufacturer's instructions. Detailed information on the enzyme‐linked immunosorbent assay (ELISA) kits is provided in the Supporting Information.

To verify the quantitative consistency between the HRM‐DIA and the immunoassay platforms, we performed a correlation analysis for six overlapping proteins (APOA1, APOA2, APOB, APOE, C3 and C4), observing a strong positive correlation between the two methods (Pearson's correlation coefficient *r* = 0.74–0.86; Figure S2), confirming the comparability of the independent datasets.

### 2.5 Data Processing for Proteomic Analysis

The spectral library from the pooled plasma in the discovery cohort and DIA data from individual samples were analysed using Spectronaut 16 (Biognosys, Schlieren, Switzerland). The FDR was set to 1% at the peptide precursor and protein levels. Default quantification settings were used with global normalization. Subsequently, missing values were imputed using random sampling from the low‐abundance signal distribution observed throughout the experiment (Table S1).

For HRM‐DIA analysis in the validation stage, the direct DIA library was generated using Pulsar embedded in Spectronaut v18 against the UniProt Human Database (July 2021, 101 014 entries) and iRT standard peptide sequence [15]. Individual DIA raw files were analysed using Spectronaut v18 with the directDIA library using the default search settings. After quantification was performed using the automatic setting and normalized with the integrated cross‐run normalization feature, unless otherwise specified, the protein abundances of the 31 targets were extracted. The detailed process of MS data processing is described in the Supporting Information.

### 2.6 Statistical Analysis

Normally distributed data are presented as the mean ± standard deviation, and categorical data are reported as a number (%). Participants characteristics in both groups were compared using the Student's *t*‐test for continuous variables and the χ^2^ test for categorical variables. Multivariable regression was performed to evaluate each biomarker and elucidate its correlation with sarcopenia criteria, adjusting for age, sex, BMI and steroid use (prednisolone 5 mg or equivalent dose for over 3 months), using a linear regression model. Nevertheless, ASM/BMI was excluded from the adjustment because it was divided by the BMI. Potential interaction terms (age × sex and sex × BMI) were tested using multivariate regression models, with no significant interactions observed. To calculate the standardized beta coefficient, the independent variables were standardized to have a mean and standard deviation of 0 and 1, respectively, before the regression model. *p* < 0.05 was regarded as statistically significant. All analyses were conducted using the R statistical software (version 4.2.1) or GraphPad Prism (version 10.0.2).

DIA data preprocessing and statistical analysis were performed using Perseus software version 1.6.15.0 [16]. A log2 transformation was applied to these values due to the skewed distribution. The preprocessed matrix was subjected to pairwise comparison based on Student's *t*‐test and Pearson's correlation analysis with the correlation parameters.

### 2.7 Bioinformatics Analysis

Canonical pathway enrichment was performed using Ingenuity Pathway Analysis (IPA) (QIAGEN, Hilden, Germany) with Fisher's exact test (*p* < 0.05), and the extent of activation was reflected by the z‐score. After the protein–protein interactions (PPI) of the selected proteins were obtained from the String version 11 database (https://string‐db.org) [17], network models were constructed using Cytoscape version 3.10 software [18].

## 3 Results

### 3.1 Baseline Characteristics of Participants

In the discovery cohort, participants were categorized into three groups based on their sarcopenia status over 2 years, as described in Section 2: those who remained nonsarcopenic (Group 1), those who developed sarcopenia (Group 2) and those who remained sarcopenic (Group 3). Groups 1 and 2 exhibited similar baseline characteristics regarding age, sex and BMI (Table 1), while Group 3 demonstrated a significantly lower proportion of female participants compared to the other groups (*p* = 0.03). ASM and HGS were higher in Group 3 than in Group 2 (both *p* < 0.01), and walking speed was similar between the groups. Furthermore, the decline in ASM and HGS over the 2‐year follow‐up was most pronounced in Group 2 (both *p* < 0.01). Physical performance, including the SPPB score and sit‐to‐stand test results, tended to be lower in Group 3 than in the other groups; nevertheless, these differences did not reach statistical significance. Similarly, no significant differences in comorbidities were observed between the groups.

**TABLE 1:** Baseline characteristics according to sarcopenia groups of discovery set.

	Group 1	Group 2	Group 3	*p*
	(*N* = 65)	(*N* = 63)	(*N* = 43)	
Female	32 (49.2%)	35 (55.6%)	13 (30.2%)	0.03^a^
Age, years	78.3 [74.6; 81.3]	77.7 [75.5; 81.1]	80.42 [76.9; 81.8]	0.07
Body mass index, kg/m^2^	23.14 ± 2.57	23.46 ± 2.84	22.70 ± 3.06	0.39
DM	18 (27.6%)	18 (28.5%)	14 (32.5%)	0.85
HTN	40 (61.5%)	39 (61.9%)	23 (53.4%)	0.63
Cardiovascular disease	2 (3.0%)	7 (10.8%)	2 (4.6%)	0.83
Cerebrovascular disease	2 (3.0%)	9 (14.2%)	2 (4.6%)	0.06
Baseline ASM, kg/m^2^	6.41 ± 1.15	6.17 ± 1.05	5.80 ± 0.74	0.01^b^, ^c^
Changes in ASM, kg/m^2^	−0.26 [−0.77; 0.01]	−0.60 [−1.02; −0.16]	−0.17 [−0.50; −0.02]	< 0.01^a^
Low muscle mass, baseline	26 (40.0%)	27 (42.8%)	43 (100.0%)	< 0.01^b^, ^c^
Baseline HGS, kg	27.7 [21.9; 32.6]	20.7 [18.8; 28.5]	23.6 [16.9; 25.4]	< 0.01^a^, ^b^
Changes in HGS, kg	0.00 [−1.0; 1.5]	−2.70 [−3.9; −1.0]	−0.2 [−1.7; 1.1]	< 0.01^a^
Low muscle strength, baseline	4 (6.1%)	20 (31.7%)	43 (100.0%)	< 0.01^a^, ^b^
Baseline SPPB, score	9.7 [8.0; 11.0]	8.8 [7.0; 11.0]	7.0 [6.0; 9.0]	0.11
Baseline walking speed, m/s	0.94 [0.81; 1.05]	0.96 [0.84; 1.12]	0.98 [0.91; 1.16]	0.09
Changes in walking speed, m/s	−0.07 [−0.16; 0.02]	−0.02 [−0.15; 0.07]	−0.00 [−0.08; 0.06]	0.10
Baseline sit‐to‐stand test, s	10.7 [8.8; 12.8]	11.3 [9.5; 12.7]	12.2 [10.0; 14.2]	0.07
Baseline low physical performance	52 (80.00%)	51 (81.95%)	33 (76.74%)	0.53

*Note:* Groups 1, 2 and 3 represent participants who stayed nonsarcopenic, who became sarcopenic and who stayed sarcopenic during 2 years of follow‐up. Data are presented as mean ± standard deviation for normally distributed variables and as median [interquartile range] for non‐normally distributed variables. For continuous variables, comparisons were conducted using Student's *t*‐test or the Mann–Whitney *U* test, as appropriate. For categorical variables, statistical analyses were performed using the chi‐square test or Fisher's exact test.

Abbreviations: ASM, appendicular skeletal muscle; DM, diabetes mellitus; HGS, handgrip strength; HTN, hypertension; SPPB, short physical performance battery.

^a^ *p* < 0.05 between Groups 1 and 2.

^b^ *p* < 0.05 between Groups 1 and 3.

^c^ *p* < 0.05 between Groups 2 and 3.

Table 2 displays a comparison between the discovery and external validation cohorts. The external validation cohort had a higher proportion of female participants (87.6% vs. 46.8%) and a younger mean age (74.3 ± 12.3 vs. 78.4 ± 3.9 years, both *p* < 0.01) compared to the discovery cohort. The baseline ASM, HGS and walking speed were significantly lower in the external validation cohort (all *p* < 0.01), along with a higher prevalence of low muscle mass, low muscle strength and low physical performance (all *p* < 0.01) than in the discovery cohort. The follow‐up duration was comparable between cohorts (24.0 vs. 23.8 months in the discovery and external validation cohorts, respectively).

**TABLE 2:** Baseline characteristics of discovery and external validation datasets.

	Discovery	External validation	*p*
	(*N* = 171)	(*N* = 93)	
Female	80 (46.8%)	78 (87.6%)	< 0.01
Age, years	78.4 ± 3.9	74.3 ± 12.3	< 0.01
Body mass index, kg/m^2^	23.1 ± 2.8	22.1 ± 4.3	0.04
DM	50 (29.24%)	9 (10.11%)	0.01
HTN	102 (59.6%)	31 (34.8%)	0.01
Cardiovascular disease	11 (6.4%)	4 (4.5%)	0.08
Cerebrovascular disease	13 (7.6%)	7 (7.8%)	0.16
Baseline ASM, kg/m^2^	6.10 [5.33; 6.82]	4.87 [4.49; 5.34]	< 0.01
Changes in ASM, kg/m^2^	−0.31 [−0.83; −0.02]	0.00 [−0.23; 0.17]	< 0.01
Low muscle mass, baseline	96 (56.1%)	73 (82.0%)	< 0.01
Baseline HGS, kg	24.4 [19.6; 28.8]	18.1 [15.8; 22.6]	< 0.01
Changes in HGS, kg	−0.8 [−2.8; 0.7]	0.30 [−2.3; 3.0]	0.04
Low muscle strength, baseline	67 (39.1%)	50 (56.1%)	< 0.01
Baseline SPPB, score	9.0 [7.0; 11.0]	9.0 [8.0; 11.0]	0.08
Baseline walking speed, m/s	0.96 [0.87; 1.10]	0.80 [0.67; 1.00]	< 0.01
Baseline five‐time sit‐to‐stand test, s	11.3 [9.3; 13.0]	15.0 [11.0; 21.0]	< 0.01
Changes in walking speed, m/s	−0.02 [−0.14; 0.05]	−0.20 [−0.33; 0.00]	< 0.01
Low physical performance, baseline	136 (79.5%)	79 (88.7%)	< 0.01
Follow‐up duration, months	24.0 [24.0; 24.0]	23.8 [21.7; 26.7]	0.481

*Note:* Groups 1, 2 and 3 represent participants who stayed nonsarcopenic, who became sarcopenic and who stayed sarcopenic during 2 years of follow‐up. Data are presented as mean ± standard deviation for normally distributed variables and as median [interquartile range] for non‐normally distributed variables. For continuous variables, comparisons were conducted using Student's *t*‐test or the Mann–Whitney *U* test, as appropriate. For categorical variables, statistical analyses were performed using the chi‐square test or Fisher's exact test.

Abbreviations: ASM, appendicular skeletal muscle; DM, diabetes mellitus; HGS, handgrip strength; HTN, hypertension; SPPB, short physical performance battery.

### 3.2 Differentially Expressed Proteins (DEPs) Among Groups

**FIGURE 2:**
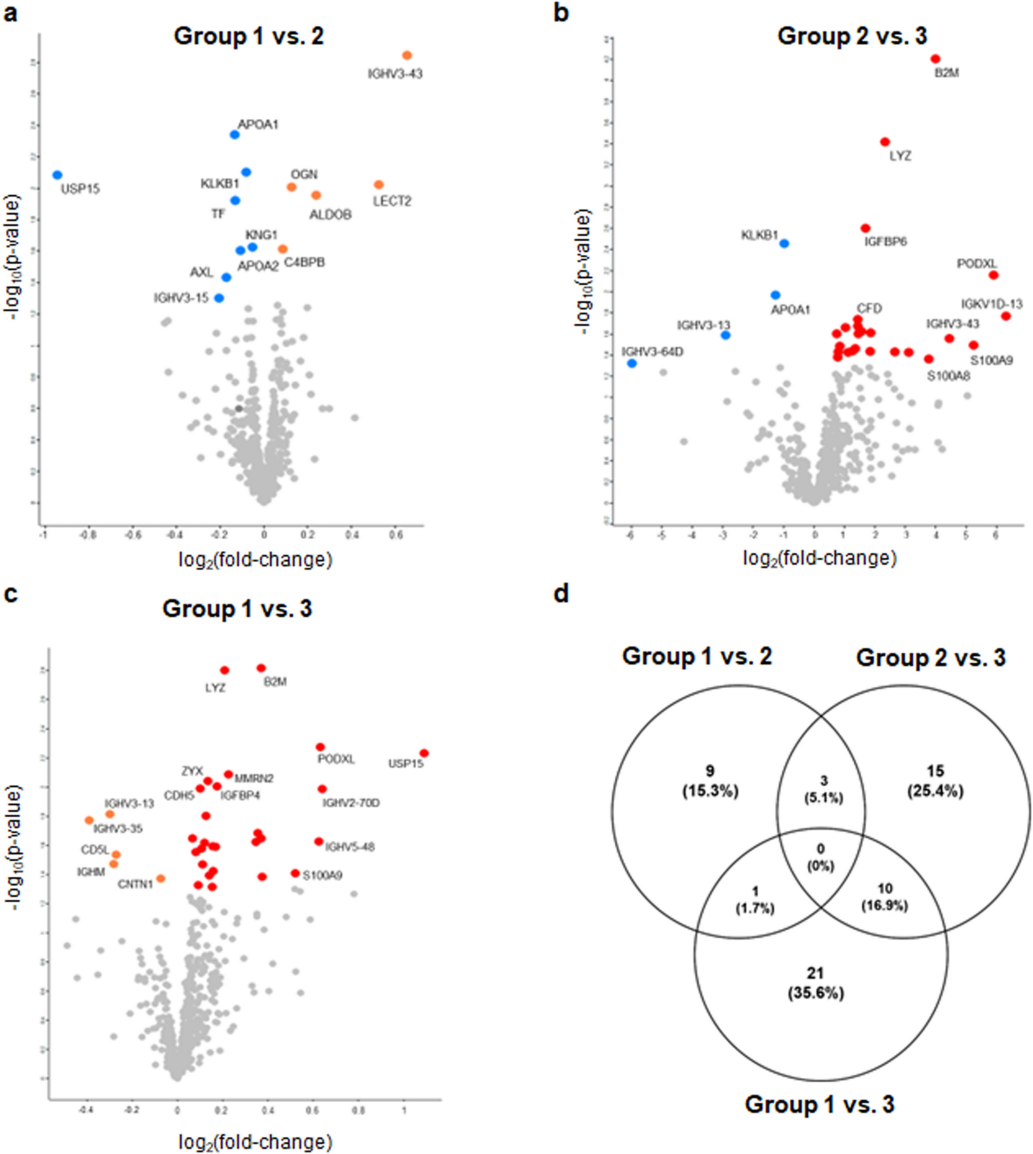
Volcano plot of pair‐wise comparison analysis. (a) Group 1 vs. Group 2, (b) Group 2 vs. Group 3, (c) Group 1 vs. Group 3 and (d) Venn diagram of overlapping DEPs among the pair‐wise comparison groups.

To investigate proteomic differences associated with sarcopenia progression, we conducted pairwise comparisons among the three groups. In the comparison between Groups 1 and 2, several proteins differed significantly (Figure 2a). USP15 was upregulated in Group 1, whereas IGHV3‐43, LECT2, ALDOB and OGN were upregulated in Group 2. Additionally, APOA1, APOA2, KLKB1 and TF differed significantly, suggesting potential involvement in sarcopenia development. In the comparison between Groups 2 and 3, B2M and LYZ were upregulated in Group 3, indicating potential roles in chronic sarcopenia (Figure 2b). Similarly, IGFBP6, PODXL, CFD and S100A8/A9 were enriched in Group 3, whereas KLKB1 and APOA1 were relatively more abundant in Group 2. In the comparison between Groups 1 and 3, B2M, LYZ, PODXL and USP15 were upregulated in Group 3, reinforcing their potential roles in persistent sarcopenia (Figure 2c). ZYX, IGFBP4, CDH5 and MMRN2 were also more abundant in Group 3, whereas IGHV3‐13, IGHM and CNTN1 were upregulated in Group 1.

The overlap of DEPs across the three pairwise comparisons (Figure 2d) demonstrated that 10 proteins (16.9%) were shared between Groups 1 and 3 and between Groups 2 and 3, suggesting common molecular signatures of sarcopenia persistence. However, no proteins were exclusively shared among the three comparisons, indicating distinct proteomic profiles at different stages of sarcopenia progression. The largest subset of unique DEPs (35.6%) was observed in the comparison between Groups 3 and 2, indicating distinct proteomic alterations in advanced sarcopenia.

### 3.3 Proteins Significantly Associated With Sarcopenia Components: Muscle Mass, Strength and Performance

**FIGURE 3:**
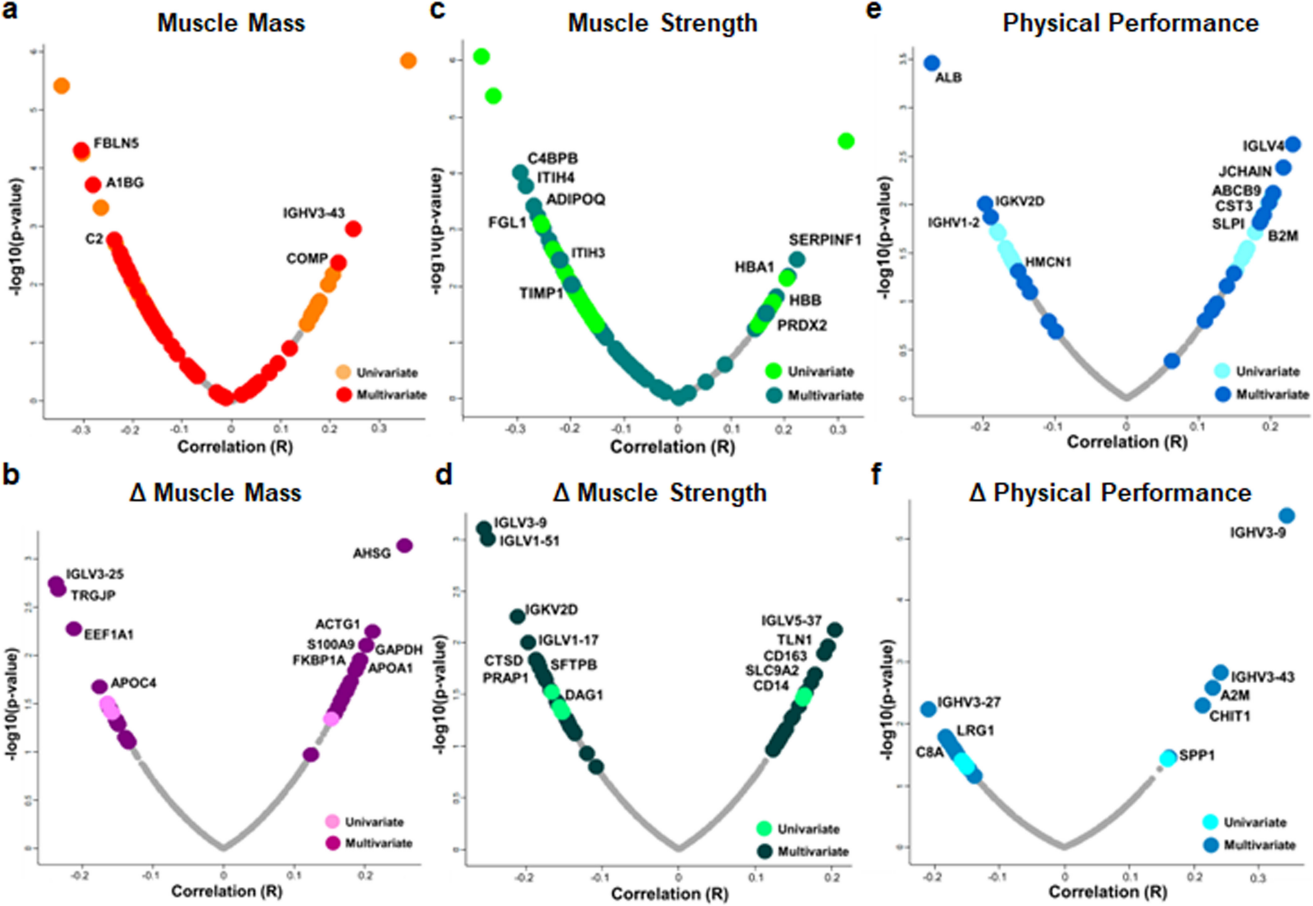
Correlation analysis between protein abundance and sarcopenic components. (a) muscle mass, (b) changes in muscle mass, (c) muscle strength, (d) changes in muscle strength, (e) physical performance and (f) changes in physical performance.

To further explore the molecular basis of sarcopenia, we examined the correlation between protein abundance and the three key sarcopenia components: muscle mass, muscle strength and physical performance (Table S1). Figure 3 presents a correlation analysis between protein expression levels and these clinical parameters. Muscle mass was analysed in both its absolute value and its change over time (Δ muscle mass). FBLN5, C2 and COMP significantly correlated with muscle mass (Figure 3a), whereas TRGJP, AHSG, S100A9 and FKBP1A were significantly associated with muscle mass changes (Figure 3b). APOA1 was also highlighted, indicating its potential metabolic involvement.

Muscle strength was assessed in both its absolute measurement and its longitudinal change (Δ muscle strength). Proteins such as C4BPB, ITIH4, ADIPOQ, FGL1, SERPINF1 and TIMP1 were significantly associated with the baseline muscle strength (Figure 3c). Moreover, CTSD, PRAP1 and SFTPB levels were significantly associated with muscle strength changes, suggesting their potential as physical performance biomarkers (Figure 3d). Physical performance was analysed similarly, using both its absolute value and its change over time (Δ performance). ALB, HMCN1, JCHAIN and B2M significantly correlated with physical performance (Figure 3e). Proteins, such as A2M and CHIT1, were associated with performance changes (Figure 3f).

**FIGURE 4:**
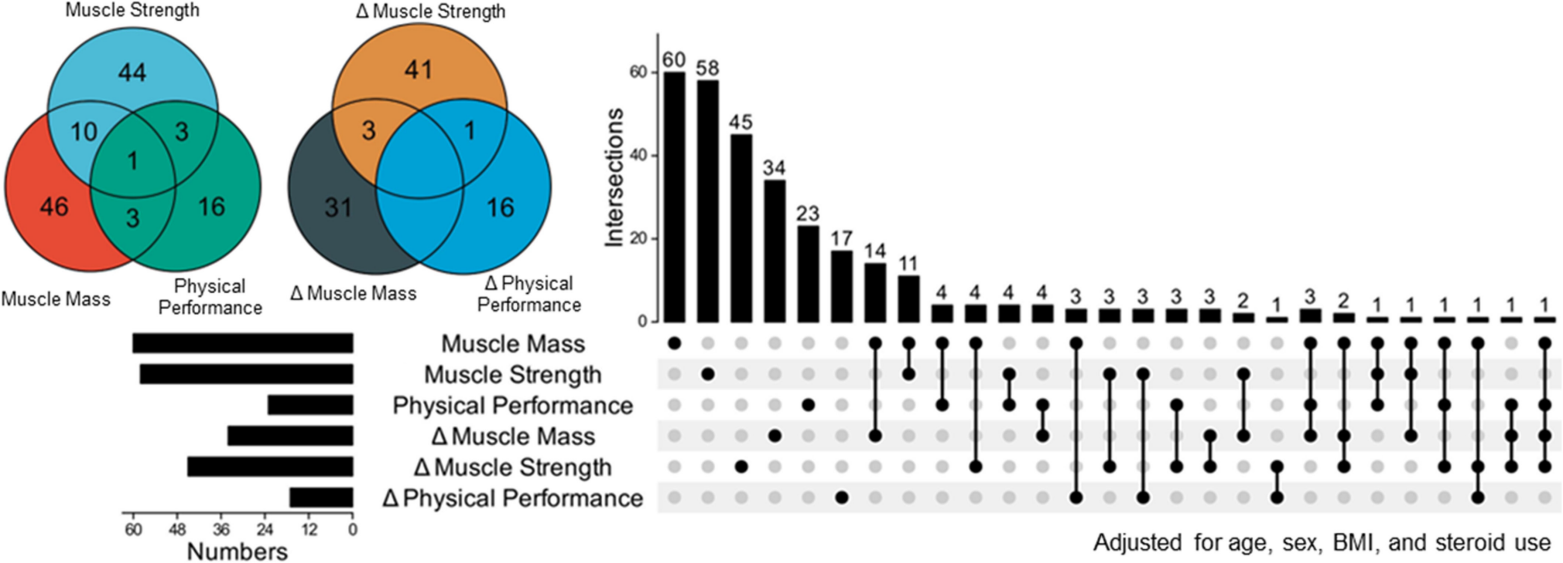
Overlap of the correlated proteins among sarcopenic components.

As displayed in Figure 4, the protein overlap was associated with muscle mass, function and performance. Most proteins were uniquely associated with a single domain, with 46 proteins (37.4%), 44 (35.8%), and 16 (13%) linked to muscle mass, muscle strength and physical performance, respectively (Figure 4). Notably, fewer than 10% of proteins overlapped across at least two components, whereas only one protein (CST3) was shared across all three. When longitudinal changes were assessed, 31 proteins (33.7%), 41 (44.6%), and 16 (17.4%) were uniquely correlated with changes in muscle mass, muscle strength and physical performance, respectively. Specifically, no proteins overlapped across any of the three longitudinal domains, underscoring the heterogeneous biological pathways underlying sarcopenia progression.

### 3.4 Key Proteins in the PPI Network Associated With Sarcopenia Components

To elucidate the molecular mechanisms underlying muscle mass, function and physical performance, we constructed PPI networks for proteins that significantly correlated with each sarcopenia‐related parameter (Figure S3). The muscle mass network identified COMP, C2, ITIH4 and C1QB as key proteins, suggesting a role for extracellular matrix remodelling and immune activation in muscle maintenance. Metabolic proteins, including PKM, PRDX2 and APOA4, were also interconnected, indicating metabolic dysregulation during sarcopenia‐related muscle loss (Figure S3A).

Regarding muscle mass changes over time, inflammation‐related proteins such as S100A8, S100A9 and ALB were prominent, along with CLU, APOA1 and ORM1, which are associated with lipid metabolism and immune responses (Figure S3B). Additionally, complement system proteins (C9 and C4BPB) were closely associated with muscle strength loss. Proteins such as ALB, CD14, CD163 and TLN1 were strongly associated with the muscle strength network, indicating a link between immune regulation, cytoskeletal stability and muscle strength. The complement system components (C5, C7, C9 and FCN3) further reinforced the role of innate immune mechanisms (Figure S3C). Proteins such as CD14, CD163, TLN1 and DAG1 were closely linked to muscle strength changes, suggesting their role in immune signalling and structural maintenance (Figure S3D).

The physical performance network revealed that ALB, B2M, FGG, CST3, ABCB9 and coagulation‐related proteins (F13B and SERPINF2) were notable (Figure S3E). Regarding performance changes over time, the key proteins were SPP2, A2M and C2, suggesting their potential roles in protease inhibition and complement activation. C8A, APOH and KNG1 were also connected, indicating that the vascular and coagulation pathways are potential contributors (Figure S3F).

When we performed a subgroup analysis by sex, we observed limited overlap in the significant proteins correlated with clinical parameters between the two sexes, indicating that distinct proteomic signatures are associated with sarcopenia in males and females (Figure S4).

### 3.5 Enriched Pathways Associated With Sarcopenia Components

The complement cascade was the most enriched pathway for muscle mass, emphasizing the role of immune system activation in muscle maintenance (Figure S5). Other significantly enriched pathways included LXR/RXR activation, glycolysis, neutrophil degranulation and integrin signalling. Additionally, IL‐12 signalling in macrophages and B‐cell receptor signalling were notable, suggesting immune‐modulated muscle homeostasis. When analysing muscle mass changes over time, pathways related to complement activation and atherosclerosis signalling were prominent (Figure S5A).

The muscle strength network was significantly enriched in complement cascade, scavenger receptor binding and immune cell communication pathways. Additional enrichment in Fc receptor signalling and vascular cell interaction pathways suggests a contribution of endothelial function to muscle integrity maintenance. Similar immune‐related pathways remained dominant for longitudinal changes in muscle strength, with notable enrichment in the complement cascade and Fc receptor pathways (Figure S5B).

Pathway enrichment for physical performance indicated distinct involvement of vascular interactions and immune modulation, including neutrophil degranulation and immune cell communication. For longitudinal performance changes, the complement cascade and platelet cytosolic calcium response were highly enriched, suggesting a potential link between immune activation, coagulation and physical decline (Figure S5C).

In a subgroup analysis by sex, pathways related to muscle mass and physical performance demonstrated significant sex differences, whereas those associated with muscle strength/change in muscle strength exhibited conserved trends across both groups (Figure S6).

### 3.6 Validation of Significant Proteins From the Discovery Cohort in the Validation Cohort

To confirm the robustness of our findings, we prioritized the identification of proteins that demonstrated consistently significant associations with sarcopenia components in both the discovery and validation cohorts. We identified seven key proteins, namely, LRG1, CST3, TIMP1, C2, ITIH1, AMBP and LYZ, which were validated with consistent directionality and statistical significance (*p* < 0.05) in both cohorts (Table S2 and Figure S7). Notably, LRG1 and TIMP1 exhibited reproducible negative correlations with muscle strength. Conversely, CST3 demonstrated a consistent positive correlation with physical performance, whereas C2 was confirmed as a significant marker negatively associated with muscle mass.

**FIGURE 5:**
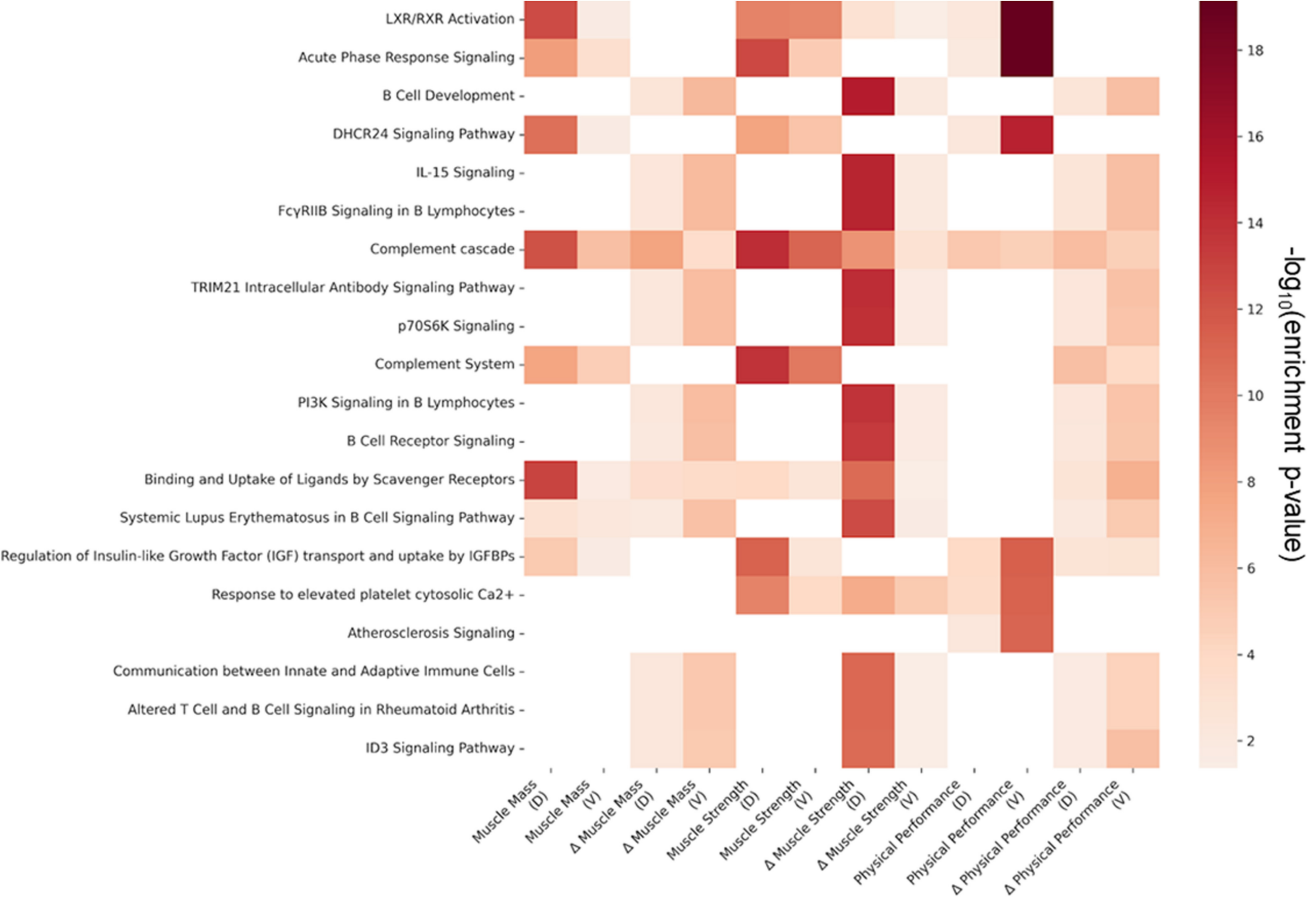
Comparative heatmap of canonical pathway enrichment between the discovery and validation cohorts. V, validation cohort; D, discovery cohort. The colour intensity represents the statistical significance [‐log10 (enrichment *p*‐value)] of pathways associated with baseline and longitudinal changes in muscle mass, strength and physical performance.

Regarding biological pathways, the comparative enrichment analysis showed strong concordance between the two cohorts (Figure 5). LXR/RXR activation and acute‐phase response signalling pathways were strongly enriched in both datasets, particularly with respect to physical performance and muscle strength. Additionally, the complement cascade demonstrated consistent enrichment, reinforcing the role of immune‐mediated mechanisms as universal drivers of sarcopenia progression.

## 4 Discussion

This study investigated proteomic biomarkers associated with sarcopenia progression in two independent prospective cohorts. High‐throughput proteomic analysis identified key pathways, including complement activation, lipid metabolism and acute‐phase response signalling, that may contribute to sarcopenia progression over time. Notably, seven robust protein signatures, including LRG1, CST3 and C2, were validated with consistent directionality in an external cohort, supporting their potential as reliable progression biomarkers. Although the primary objective was sarcopenia progression evaluation, secondary analyses examined whether specific proteins were differentially associated with declines in muscle mass, muscle strength and physical performance. Pathway enrichment analysis identified LXR/RXR signalling, acute‐phase response and complement cascade activation as central mechanisms across these domains, supporting mechanistic heterogeneity in the biological drivers of individual sarcopenia components.

The current findings highlight lipid metabolism, particularly LXR/RXR signalling, as a key regulator of muscle mass and performance in both the discovery and validation cohorts. Although this pathway has been primarily studied in cholesterol homeostasis, emerging evidence suggests that lipid transport dysregulation contributes to muscle physiology [19, 20, 21, 22]. LXR and RXR are nuclear receptors that regulate genes involved in lipid metabolism, including ATP‐binding cassette transporters, such as ABCA1 and ABCG1 [19]. LXR/RXR activation enhances cholesterol efflux in various cell types, thereby maintaining lipid homeostasis [20]. While most existing research has focused on tissues, such as the liver and intestines [20], emerging evidence suggests that lipid transporters regulated by LXR/RXR are crucial for muscle strength and regeneration [21, 23]. Notably, Lim et al. conducted a comparative plasma proteomics study on murine models of cancer cachexia and disuse‐induced atrophy, revealing that lipid transporter proteins involved in the LXR/RXR pathway were significantly repressed in cachectic mice, suggesting a critical role of lipid metabolism in muscle atrophy [24]. Additionally, recent metabolomic and lipidomic data in sarcopenic older adults [25] demonstrated decreased TCA‐cycle intermediates, altered amino acid and unsaturated fatty acid metabolism and increased indole and bile‐acid derivatives; our proteomic results reinforce these findings and support a shared metabolic–immune axis in sarcopenia. Specifically, the dysregulation of LXR/RXR signalling and complement activation observed in our cohorts paralleled impaired lipid oxidation and low‐grade inflammation, suggesting that disrupted lipid–immune homeostasis represents a convergent mechanism underlying muscle decline.

Complement activation was another consistently enriched pathway, with proteins such as C2, C7, C9 and C4BPB validated in relation to sarcopenia progression. These proteins were also validated as biomarkers associated with changes in muscle mass, function and physical performance. Although limited literature directly links these four factors to muscle health, several studies indicate close associations between complement pathway‐related factors and muscle health. Specifically, C1q, a key activator of the classical pathway, reportedly shows increased gene expression with ageing [26], which in turn activates the Wnt signalling pathway and contributes to muscle fibrosis [27]. Furthermore, a cross‐sectional study reported a negative correlation between serum C1q levels and muscle mass and strength [28]. CRP, a protein that activates the complement system, has also been associated with lower muscle strength and mass in the Hertfordshire Cohort [29] and the Longitudinal Aging Study Amsterdam cohort [30]. Given the emerging role of complement‐related inflammation in sarcopenia, these findings suggest that targeting complement activation may represent a novel therapeutic strategy for muscle preservation in ageing populations.

Although the baseline characteristics differed between the community‐based discovery cohort and the clinically enriched validation cohort, this heterogeneity reflects the broader clinical spectrum of sarcopenia, from generally healthy older adults to those with comorbid osteoporosis. Nevertheless, the key protein–pathway associations, especially those related to complement activation and lipid metabolism, were directionally consistent across cohorts, suggesting that they capture the fundamental biological processes underlying sarcopenia progression. This cross‐setting reproducibility enhances external validity, although residual confounding due to the cohort composition cannot be completely ruled out.

Study strengths included the longitudinal design with external validation, which enhances biomarker discovery reliability and generalizability. High‐throughput LC‐MS/MS proteomics enabled an unbiased and in‐depth molecular characterization of sarcopenia progression and persistence. Unlike prior studies, this research distinguished muscle mass, function and physical performance, revealing distinct biological pathways underlying each component. Key biomarkers, including complement system proteins, lipid transport regulators and immune‐related factors, were validated in an independent cohort, reinforcing clinical relevance. Integration of rigorous statistical analyses, PPI network modelling and pathway enrichment supported biological significance. Moreover, the findings link sarcopenia with systemic ageing mechanisms, including complement activation, lipid metabolism and immune regulation, highlighting potential targets for early detection, risk stratification and therapeutic intervention in sarcopenia management.

However, this study had some limitations. First, the sample size was modest, potentially limiting statistical power. Second, batch effects and technical variability in LC‐MS/MS analysis may have influenced quantification despite rigorous quality control. Third, proteomic analysis relied on a bottom‐up approach using neat plasma. Although this strategy prioritizes quantitative robustness, the wide dynamic range of the plasma proteome likely precludes detection of specific low‐abundance bioactive peptides (e.g., apelin or iAM373) [31], as confirmed by our retrospective screening. To compensate for this limitation and ensure coverage of established biological pathways, we concurrently measured a comprehensive panel of known sarcopenia‐related markers, including myostatin, irisin and IGF‐1, using targeted immunoassays. Fourth, the findings were observational, requiring functional validation to confirm their causality. Fifth, the cohort was limited to Asian populations, which restricts its generalizability. Sixth, the validation cohort was derived from a clinical cohort of individuals with osteoporosis, which may not fully represent the general older adult population with sarcopenia. Additionally, the follow‐up duration may not have captured long‐term sarcopenia trajectories, and further studies are warranted. Finally, although the current findings do not establish a direct causality, they provide biologically plausible leads, particularly those involving complement activation and lipid metabolism. Future in vitro and in vivo functional studies are warranted to determine whether the complement components or lipid transport proteins actively modulate muscle atrophy and regeneration. It would be valuable to establish primary muscle cell models derived from aged human skeletal muscle tissues, as well as senescence‐induced 2D/3D culture systems, to enable mechanistic evaluation of complement factors (C2 and C1R) and apolipoproteins (APOA1 and APOE) in muscle ageing and systemic inflammation.

In conclusion, this study identified and validated the circulating proteins and pathways associated with sarcopenia progression, with complement activation and lipid metabolism emerging as key mechanisms. These findings provide a foundation for the early diagnosis, risk stratification and therapeutic targeting of sarcopenia. Further mechanistic and multi‐ethnic validation studies are required to establish the clinical utility of these biomarkers.

## Funding

This study was funded by grants from the Korea Health Technology R&D Project through the Korean Health Industry Development Institute (KHIDI), supported by the Ministry of Health and Welfare, Republic of Korea (grant number: HI15C3153); the National Institute of Health (NIH) research project (project No. 2024‐ER0603‐01); National Research Foundation of Korea, Republic of Korea (grant no. 2024‐00340798, 2021R1A2C2003410, RS‐2024‐00456173, RS‐2020‐NR049540, and RS‐2023‐00220894); Seoul National University Bundang Hospital (grant no. 18‐2023‐0003); and the Korean Endocrine Society of Convergence Research Award 2023.

## Conflicts of Interest

The authors declare no conflicts of interest.

## Supporting information


**Figure S1:** Quality control of proteomic data. (A) Profile plots of total LC‐MS/MS, (B) PCA plot. LC‐MS/MS, liquid chromatography‐mass spectrometry/mass spectrometry; PCA, principal component analysis.
**Figure S2:** Correlation plot between LC‐MS/MS and ELISA method. LC‐MS/MS, liquid chromatography‐mass spectrometry/mass spectrometry; ELISA, enzyme‐linked immunosorbent assay. Scatter plots illustrating the quantitative consistency between the two analytical platforms used in the validation cohort. The x‐axis represents the log‐transformed protein concentrations measured by ELISA, and the y‐axis represents the log‐transformed protein intensities measured by HRM‐DIA MS.
**Figure S3:** Protein–protein interaction (PPI) networks of the proteins correlated with sarcopenic components. (A) muscle mass, (B) changes in muscle mass, (C) muscle strength, (D) changes in muscle strength, (E) physical performance and (F) changes in physical performance.
**Figure S4:** Distinct proteomic signatures according to sex. The number of overlapping proteins correlated with sarcopenia components (muscle mass, strength and physical performance) between males and females are presented.
**Figure S5:** Enrichment analysis based on canonical pathway. (A) Baseline and longitudinal changes of muscle mass, (B) function and (C) performance. Analysis was done by Ingenuity Pathway Analysis (IPA).
**Figure S6:** Comparison of enriched canonical pathways between sexes. Pathway enrichment analysis was performed separately for male and female participants in the discovery cohort to identify sex‐specific biological mechanisms. The heatmaps display the top canonical pathways associated with (left panel) muscle mass and its longitudinal change, (middle panel) muscle strength and its change and (right panel) physical performance and its change. The colour intensity represents the activation z‐score, with orange indicating pathway activation and blue indicating inhibition. While pathways related to muscle mass and physical performance show significant divergence between sexes, pathways associated with muscle strength exhibit relatively conserved trends across both groups.
**Figure S7:** Heatmap of the correlated proteins in the validation set. Heatmap displaying the correlation coefficients (R) of key proteins identified in the discovery cohort with sarcopenia components in the independent validation cohort. The colour scale indicates the strength and direction of the correlation (red: positive, blue: negative).

**Table S1:** Results of correlation analysis in discovery cohort.
**Table S2:** Results of correlation analysis in validation cohort.

**Data S1:** Supporting information.

## Data Availability

The mass spectrometry proteomics data have been deposited to the ProteomeXchange Consortium via the PRIDE partner repository with the dataset identifiers [PXD070542] and [PXD072647].
