# Supplementary material for: Distinct Proteomic Signatures Driving Progression of Sarcopenia: A Longitudinal Multicohort Study
Source: J Cachexia Sarcopenia Muscle. 2026 Mar 4;17(2):e70240. doi: 10.1002/jcsm.70240 (PMC12961230; doi:10.1002/jcsm.70240)
Supplement: Supplementary file 1 — Figure S1: Quality control of proteomic data. (A) Profile plots of total LC‐MS/MS, (B) PCA plot. LC‐MS/MS, liquid chromatography‐mass spectrometry/mass spectrometry; PCA, principal component analysis. Figure S2: Correlation plot between LC‐MS/MS and ELISA method. LC‐MS/MS, liquid chromatography‐mass spectrometry/mass spectrometry; ELISA, enzyme‐linked immunosorbent assay. Scatter plots illustrating the quantitative consistency between the two analytical platforms used in the validation cohort. The x‐axis represents the log‐transformed protein concentrations measured by ELISA, and the y‐axis represents the log‐transformed protein intensities measured by HRM‐DIA MS. Figure S3: Protein–protein interaction (PPI) networks of the proteins correlated with sarcopenic components. (A) muscle mass, (B) changes in muscle mass, (C) muscle strength, (D) changes in muscle strength, (E) physical performance and (F) changes in physical performance. Figure S4: Distinct proteomic signatures according to sex. The number of overlapping proteins correlated with sarcopenia components (muscle mass, strength and physical performance) between males and females are presented. Figure S5: Enrichment analysis based on canonical pathway. (A) Baseline and longitudinal changes of muscle mass, (B) function and (C) performance. Analysis was done by Ingenuity Pathway Analysis (IPA). Figure S6: Comparison of enriched canonical pathways between sexes. Pathway enrichment analysis was performed separately for male and female participants in the discovery cohort to identify sex‐specific biological mechanisms. The heatmaps display the top canonical pathways associated with (left panel) muscle mass and its longitudinal change, (middle panel) muscle strength and its change and (right panel) physical performance and its change. The colour intensity represents the activation z‐score, with orange indicating pathway activation and blue indicating inhibition. While pathways related to muscle mass and physical perf [file JCSM-17-e70240-s003.pptx]

## Slide 1
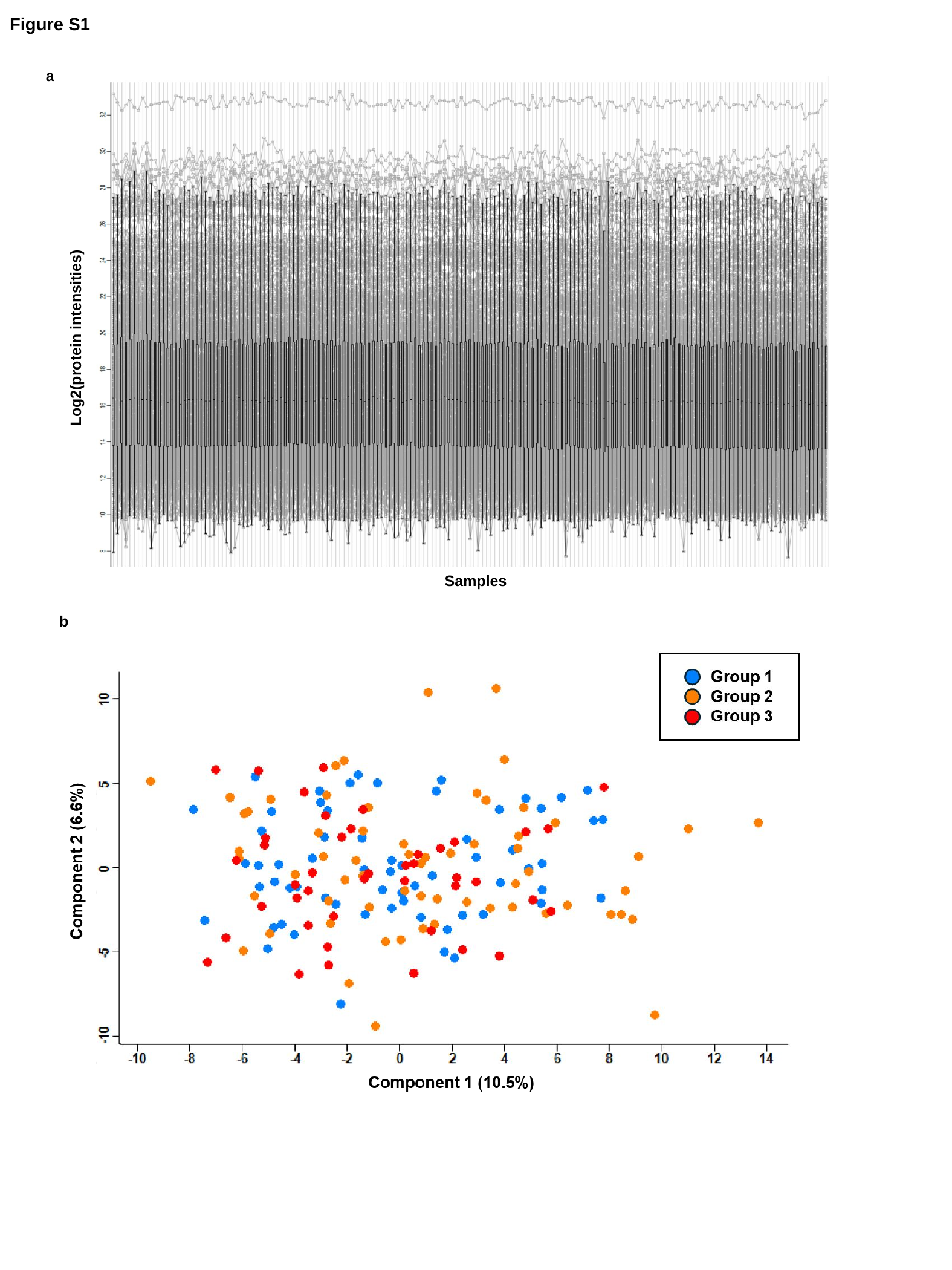

Figure S1
a
Log2(protein intensities)
Samples
b

## Slide 2
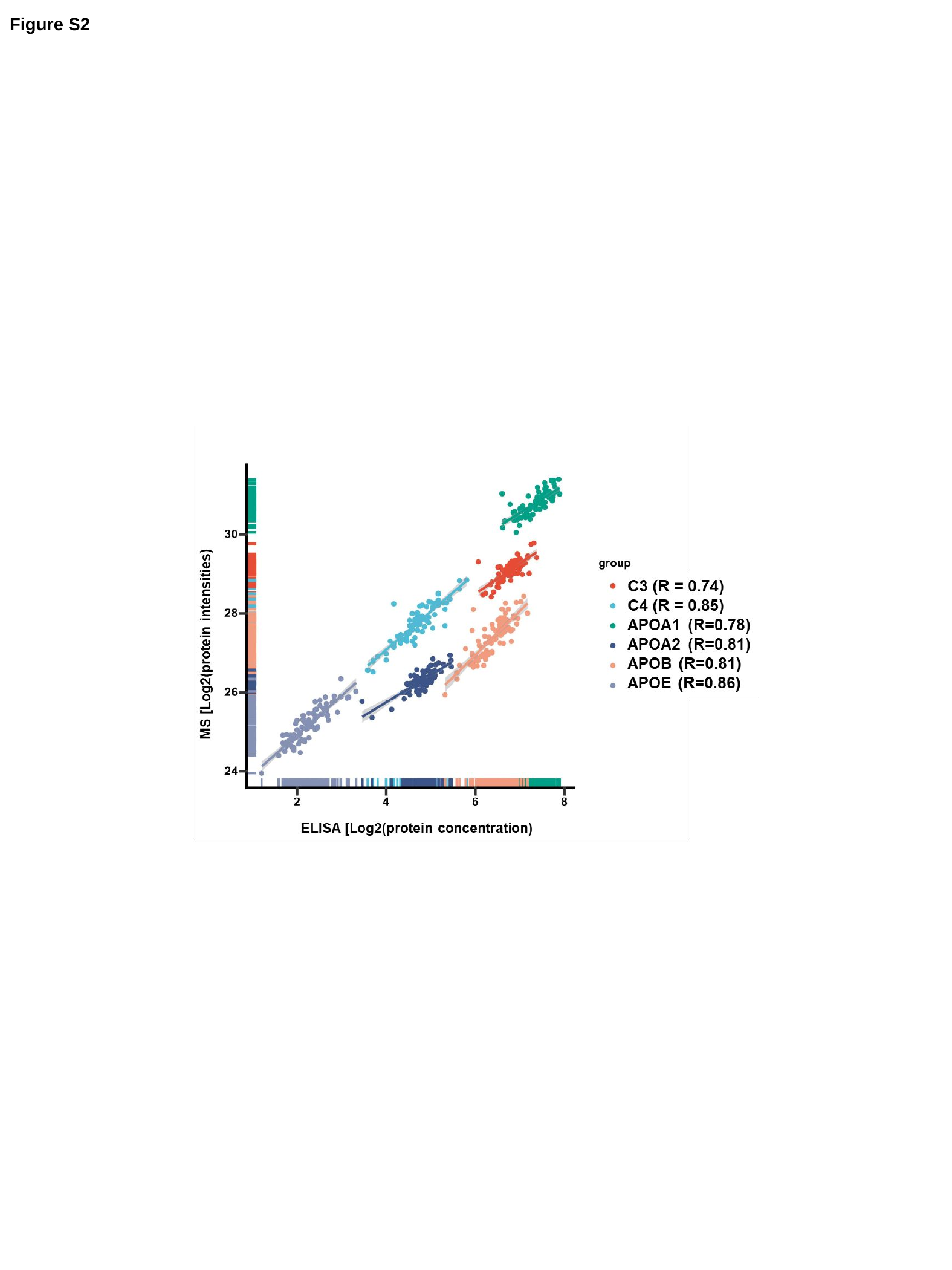

Figure S2

## Slide 3
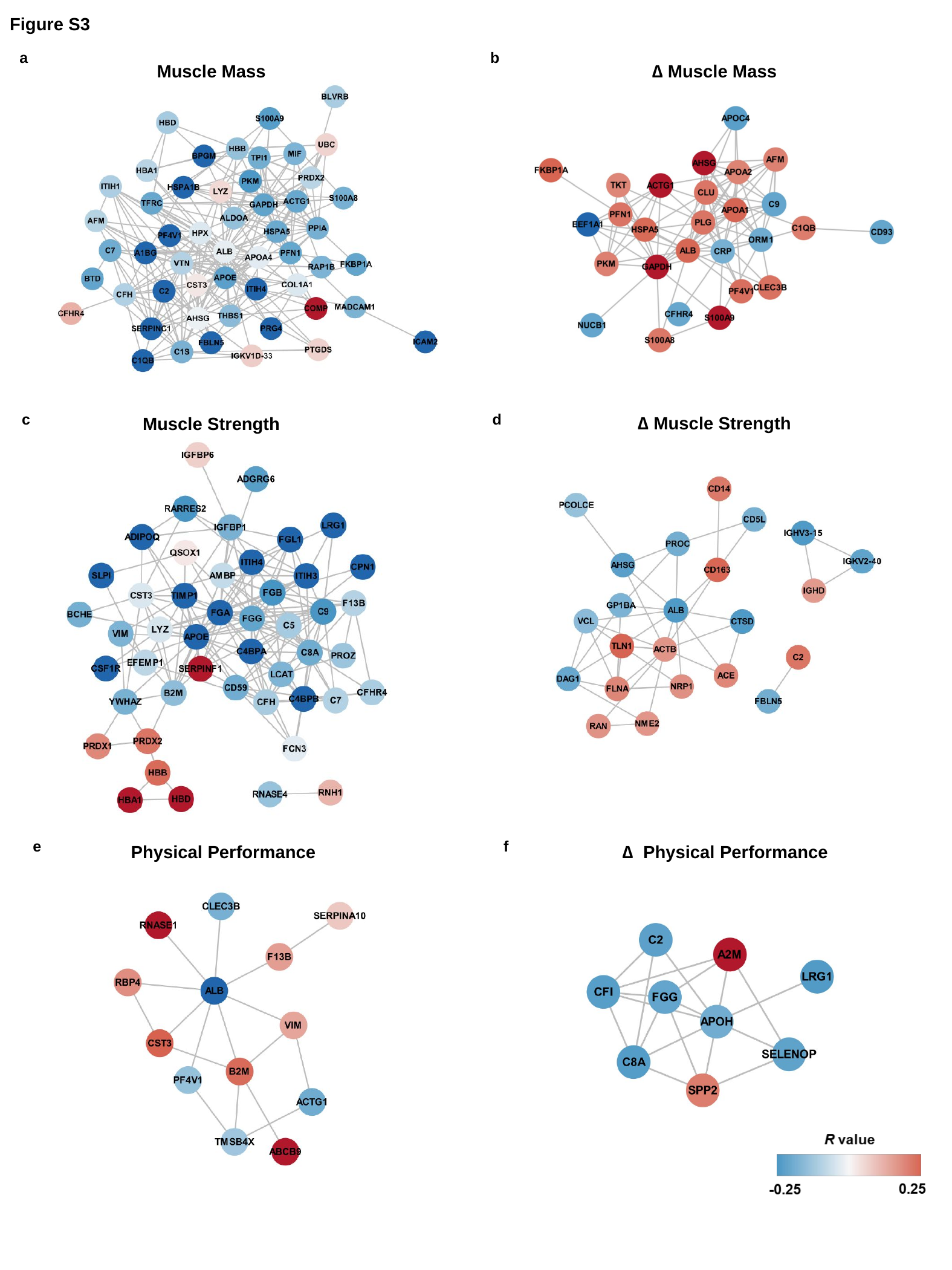

Figure S3
a
b
∆ Muscle Mass
Muscle Mass
c
d
∆ Muscle Strength
Muscle Strength
e
f
Physical Performance
∆ Physical Performance

## Slide 4
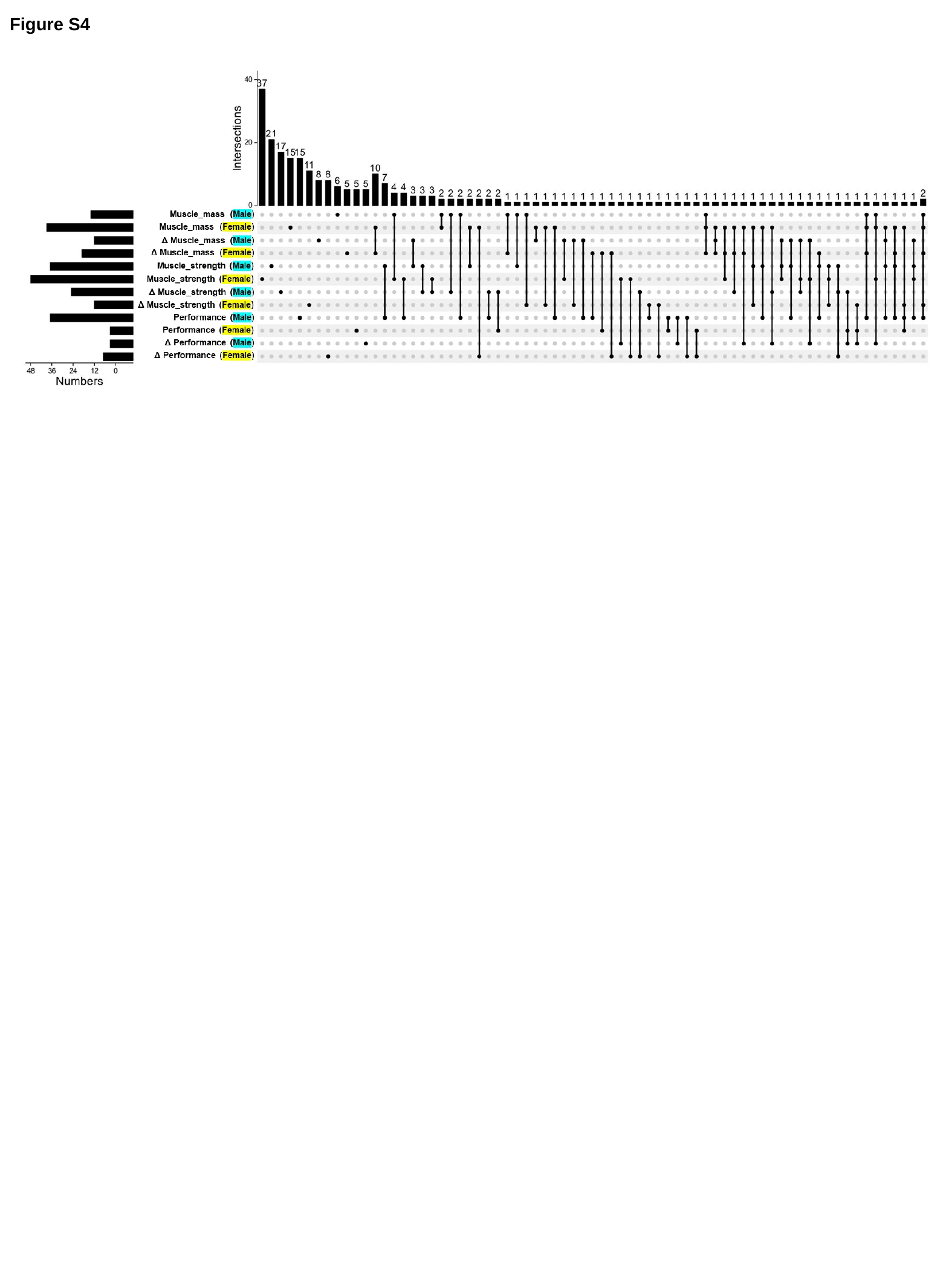

Figure S4

## Slide 5
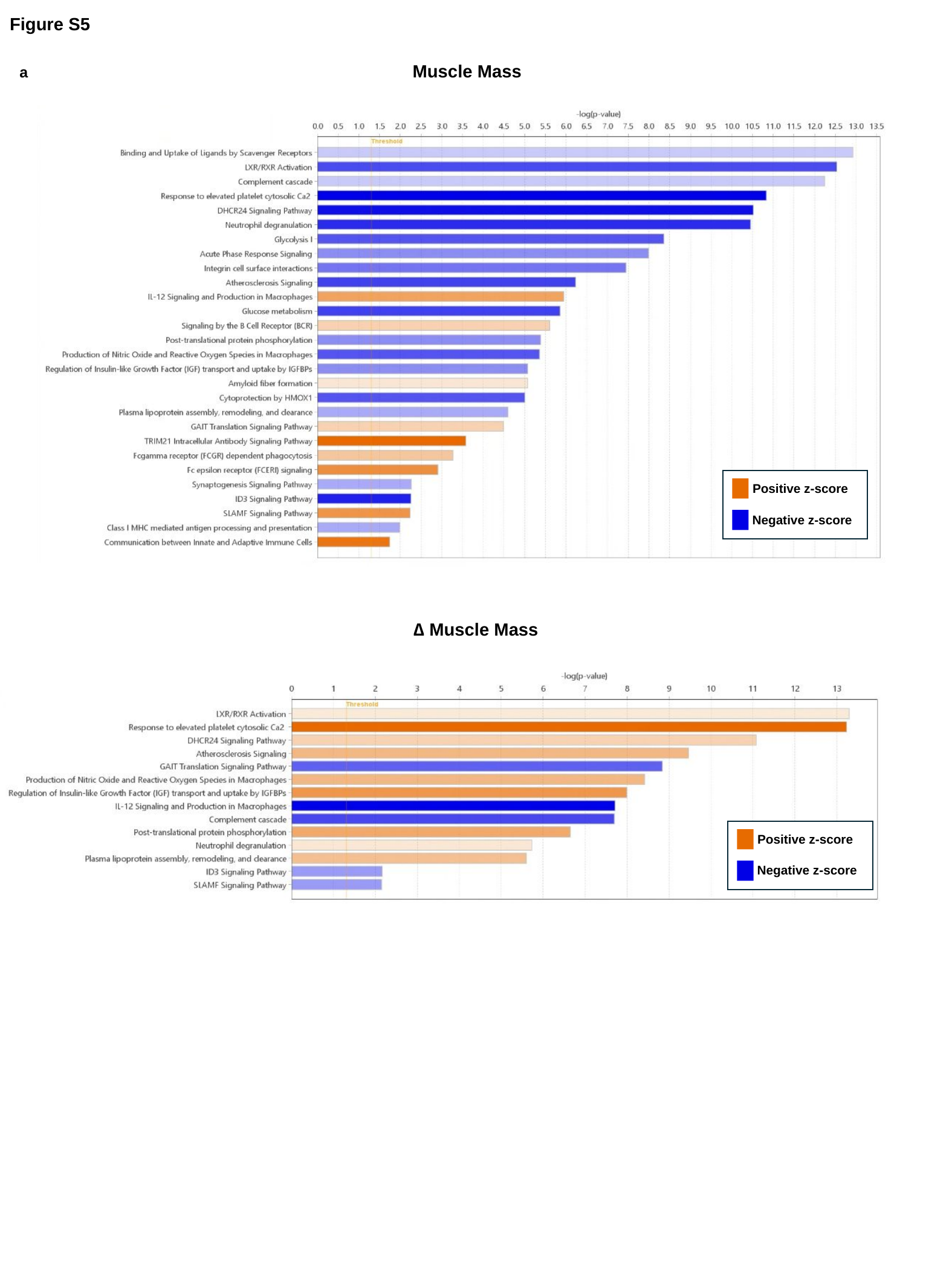

Figure S5
Muscle Mass
a
Positive z-score
Negative z-score
∆ Muscle Mass
Positive z-score
Negative z-score

## Slide 6
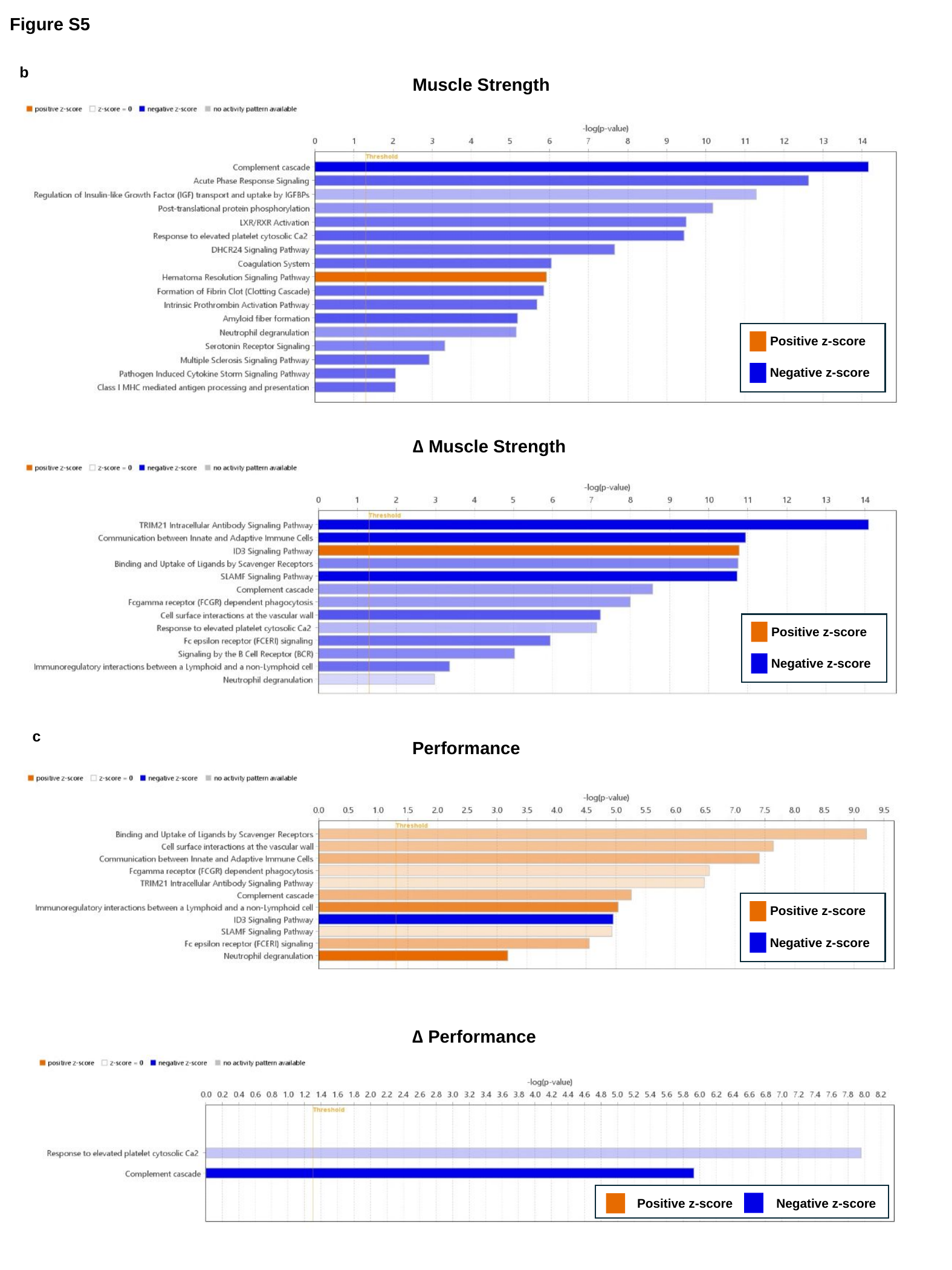

Figure S5
b
Muscle Strength
Positive z-score
Negative z-score
∆ Muscle Strength
Positive z-score
Negative z-score
c
Performance
Positive z-score
Negative z-score
∆ Performance
Negative z-score
Positive z-score

## Slide 7
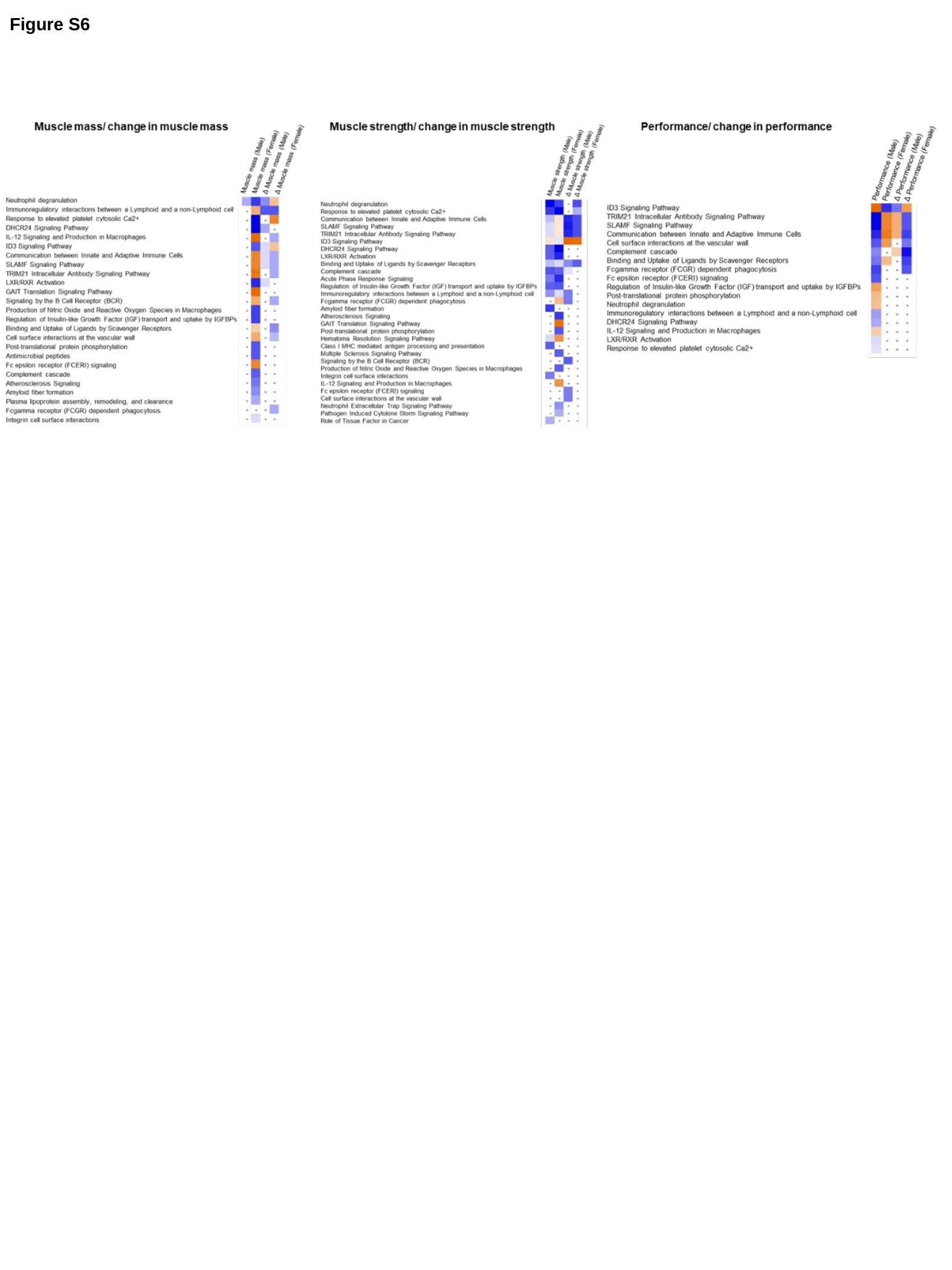

Figure S6

## Slide 8
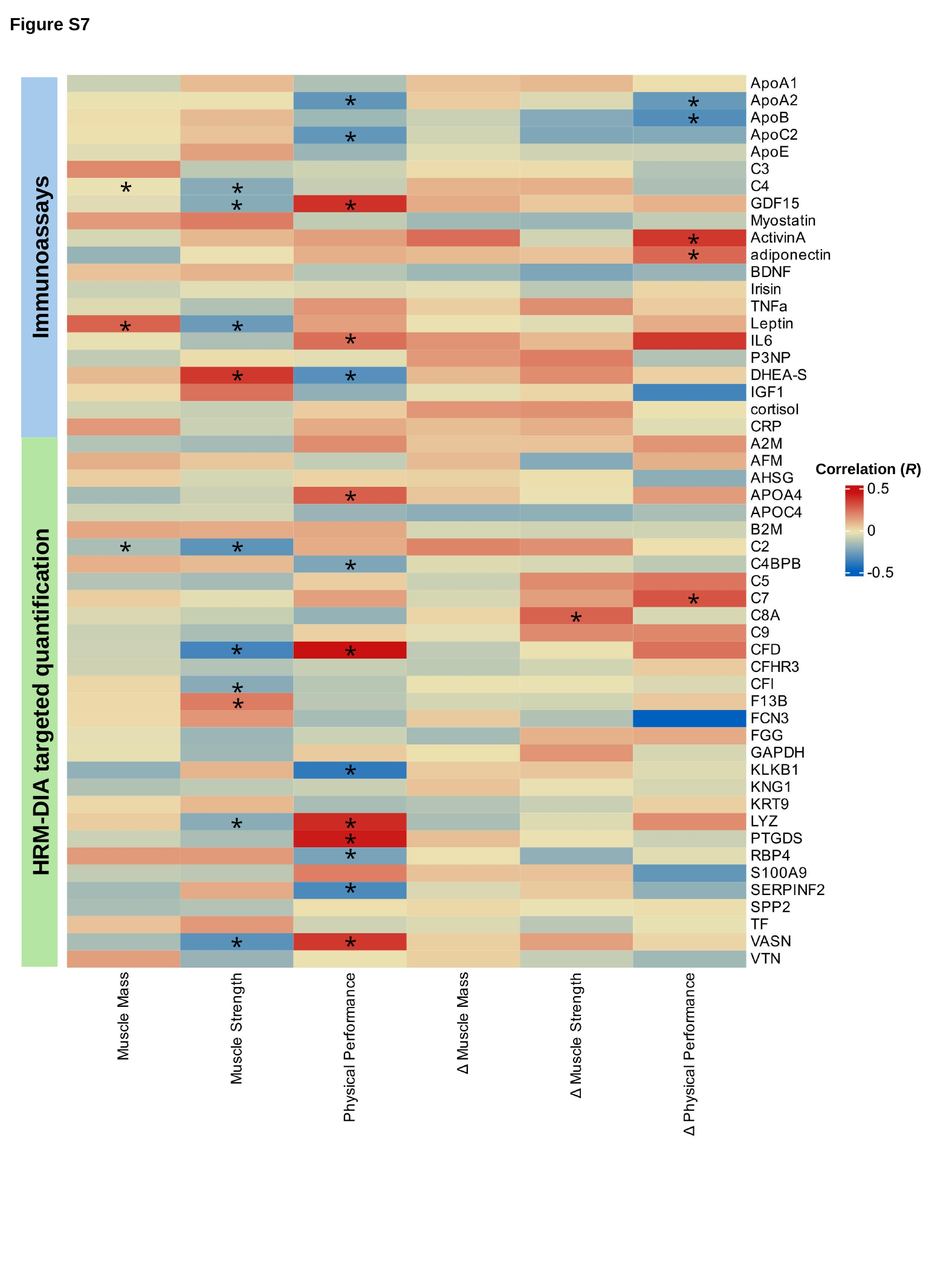

Figure S7
*
*
*
*
*
*
*
*
*
Immunoassays
*
*
*
*
*
*
Correlation (R)
*
*
*
*
*
*
*
*
*
HRM-DIA targeted quantification
*
*
*
*
*
*
*
*
*
