# Supplementary material for: Distinct Proteomic Signatures Driving Progression of Sarcopenia: A Longitudinal Multicohort Study
Source: J Cachexia Sarcopenia Muscle. 2026 Mar 4;17(2):e70240. doi: 10.1002/jcsm.70240 (PMC12961230; doi:10.1002/jcsm.70240)
Supplement: Supplementary file 3 — Data S1: Supporting information. [file JCSM-17-e70240-s001.docx]

**Sample preparation for proteomic analysis**

The protein digestion was performed according to one-step protein digestion as previously described with some modification [1]. Plasma samples (2 ul) were denaturated and alkylated with digestion buffer [8M UREA, 5mM tris(2-carboxyethyl) phosphine (TCEP), 20mM 2-chloroacetamide (CAA), and 0.1M Tris pH 8.5 for 30min at 60 °C. After cooling to room temperature, proteins were digested at 37 °C overnight using a trypsin/LysC mixture at 100:1 protein-to-protease ratio. The additional digestion was performed at 37 °C for 2 h using trypsin (enzyme-to-substrate ratio [w/w] of 1:1000). All resulting peptides were acidified with 10% trifluoroacetic acid (TFA). The acidified peptides were loaded onto homemade C18-sulfonated styrene-divinylbenzene polymer (C18-SDB-RPS) StageTips (3 M, St. Paul, MN, USA) following previously described procedures [2]. The desalted peptides were completely dried in a vacuum dryer and stored at –80 °C until further analysis.

**Proteomic analysis**

For discovery cohorts, data-dependent acquisition (DDA) and data-independent acquisition (DIA) methods, were conducted with an Ultimate 3000 UHPLC system (Dionex, Sunnyvale, CA) coupled to a Q-Exactive Plus mass spectrometer (Thermo Fisher Scientific Inc., Waltham, MA, USA) as previously described with some modifications [3]. Peptide samples were separated on a two-column setup with a trap column (300 μm I.D. × 5 mm, C18 3 μm, 100 Å) and an analytical column (75 μm I.D. × 50 cm, C18 1.9 μm, 100 Å). Prior to sample injection, the dried peptide samples were re-dissolved in solvent A (2% acetonitrile and 0.1% formic acid). After the samples were loaded onto the nano liquid chromatography (LC), a 90-min gradient from 8 to 30% solvent B (100% acetonitrile and 0.1% formic acid) was applied to all samples. For the DDA method for spectral library generation, a survey scan (350 to 1,650 m/z) was acquired with a resolution of 70,000 at m/z 200. The top-20 method was used to select the precursor ion with an isolation window of 1.2 m/z. The tandem mass spectrometry (MS/MS) spectrum was acquired at a higher-energy collisional dissociation (HCD)-normalized collision energy of 30 with a resolution of 17,500 at m/z 200. The maximum ion injection times for the full and MS/MS scans were 20 and 100 ms, respectively. For DIA analysis, the instrument was operated in DIA mode and full scan spectra precursor spectra (350–1600 Da) were acquired with a resolution of 60,000 at m/z 200, a normalized automatic gain control (AGC) target of 300%, with a maximum injection time of 25 ms. Fragment spectra were recorded in profile mode fragmenting 41 dynamic windows covering the mass range 350 to 1200 Da with a resolution of 15,000. Isolated precursors were fragmented in the HCD cell using 30% normalized collision energy, a normalized AGC target of 1000%, and a maximum injection time of 22 ms.

For validation cohorts, the mass spectrometry analyses were performed utilizing Orbitrap Exploris 480 coupled with an Ultimate 3000 RSLC system (Dionex, Sunnyvale, CA, USA), which consisted of IonOptick Aurora LC columns (IonOpticks, Victoria, AUS). The peptide samples were subjected to separation using a two-column system. This system comprised of a trap column (300 µm I.D. × 0.5 cm, C18 3 µm, 100 Å), and an analytical column (75 µm I.D. × 15 cm, C18 1.7 µm, 120 Å). The separation process involved a 30-minute gradient from 8% to 30% Solvent B (composed of 80% acetonitrile and 0.1% formic acid) at a flow rate of 780 nL/min. The temperature of the column heater was set to 60°C. In positive mode, the spray voltage was set to 2.0 kV, while the heated capillary temperature was set to 320°C. For the Hyper Reaction Monitoring (HRM) DIA experiments, the full scan method implied scan ranges of 350–1,600 m/z with a resolution of 60,000 and an AGC target of 3 × 106 at a 25 ms injection time. The DIA scan consisted of 41 DIA isolation windows and was acquired at a resolution of 15,000. An automatic injection time with AGC target of 1 x 106 was applied.

The following proteins, which could not be quantified by mass spectrometry due to the absence of pre-established reference peptides for quantification—Apolipoprotein A-1 (APOA1), Apolipoprotein A-II (APOA2), Apolipoprotein B (APOB), Apolipoprotein C-II (APOC2), Apolipoprotein E (APOE), Complement C3 (C3), Complement C4 (C4), DHEA-S, IGF-1, Cortisol, myostatin, activin A, adiponectin, brain-derived neurotrophic factor, irisin, tumor necrosis factor alpha, leptin, interleukin-6 (IL-6), procollagen type III N-terminal peptide, and high-sensitivity C-reactive protein (hs-CRP) —were measured using ELISA. Measurements were performed according to the manufacturers’ instructions using the following kits: APOA1 (Roche, Cobas6000 c501, 3032566122, Germany), APOA2 (Sekisui, Toshiba, Apo A II Auto N Daiichi, 203RCU, Japan), Apolipoprotein B (Roche, Cobas6000 c501, 3032574122, Germany), APOC2 (Sekisui, Toshiba, Apo C II Auto N Daiichi, 204REU, Japan), APOE (Sekisui, Toshiba, Apo E Auto N Daiichi, 202RBU, Japan), Complement C3 (Roche, Cobas6000 c501, 3001938322, Germany), Complement C4 (Roche, Cobas6000 c501, 3001962322, Germany), DHEA-S (Siemens, Immulite 2000, L2KDS2, USA), IGF-1 (Roche, cobas e801, 7475918, Germany), Cortisol (Abbott, Alinity, 08P3320, Ireland), myostatin (R& D systems, DGDF80), activin A (R& D systems, DAC00B), adiponectin (Millipore, EZHADP-61K), brain-derived neurotrophic factor (R&D systems, DBNT00), irisin (Adipogen, AG-45A-0046YEK-KI01), tumor necrosis factor alpha (R&D systems, HSTA00E), leptin (Millipore, EZHL-80SK), IL-6 (R&D systems, HS600C), procollagen type III N-terminal peptide (Aviva System Biology, OKEH00548), and hs-CRP (Roche, 5950864).

**Data processing for proteomic analysis**

To generate the spectral library, 24 DDA measurements were performed with pooled plasma samples in discovery cohort. The DDA spectra were searched using Spectronaut Pulsar against the Uniprot Human Database (July 2021, 101,014 entries) and the iRT standard peptide sequence. A spectral library was generated using the spectral library generation feature of Spectronaut 16. The DIA data from individual samples were analyzed with Spectronaut 16 (Biognosys, Schlieren, Switzerland). First, we converted the DIA raw files into htrm format using the GTRMS converter tool provided with Spectronaut. The false discovery rate (FDR) was estimated with the mProphet [4] approach and set to 1% at the peptide precursor and protein levels. Proteins were inferred using the software, and quantification information was acquired at the protein level using the *q-*value < 0.01 criteria, which was used for subsequent analyses. Default settings of quantification were applied, with global normalization enabled. Spectronaut incorporates a robust normalization algorithm that corrects for systematic variations in signal intensity across different LC-MS runs. This global normalization process aims to equalize the overall protein abundance across all samples, mitigating technical variations introduced during sample handling, LC separation, and mass spectrometry analysis. Subsequently, missing values were imputed based on a random sampling from a distribution of low abundant signals taken across the entire experiment (Supplementary Table 1).

For HRM-DIA analysis in validation stage, directDIA library was generated using Pulsar embedded in Spectronaut v18 from DIA raw files of validation cohort the Uniprot Human Database (July 2021, 101,094 entries) and the iRT standard peptide sequence [5]. The settings for Pulsar and library generation were as follows: Trypsin/P as specific enzyme; peptide length from 6 to 52; max missed cleavages 2; Carbamidomethyl on C as fixed modification; Oxidation on M and Acetyl at protein N-terminus as variable modifications. FDRs at peptide-spectrum match (PSM), peptide and protein level all set to 0.01. Individual DIA raw files were analyzed using Spectronaut v19 with directDIA library and in-silico library of the spiked heavy peptides using default search settings, except for setting the "Multi-channel Workflow Definition" to "From Library Annotation" with the "Fallback Option" "Labeled" [6]. After quantification was performed using the automatic setting and normalized with the integrated cross-run normalization feature unless otherwise specified, protein abundance of 31 targets were extracted.

**Statistical analysis**

Normally distributed data was presented as the mean ± standard deviation, and categorical data was reported as number (%). Characteristics of participants in both groups were compared using Student’s t test continuous variables and the χ^2^ test for categorical variables. Multivariable regression was performed to evaluate each biomarker for elucidating correlation with sarcopenia criteria for adjustment of age, sex, and BMI based on linear regression model. However, in the case of ASM/BMI, it was excluded from the adjustment because it is already divided by BMI. To calculate the standardized coefficient beta, the independent variables were standardized to have a mean of zero and a standard deviation of one before the regression model. *P*-value below 0.05 was regarded as statistically significant. All analyses were conducted using R statistical software (version 4.2.1) or GraphPad Prism (version 10.0.2)

Preprocessing and statistical analysis of the DIA data were performed using Perseus software version 1.6.15.0 [7]. Log2 transformation was conducted for these values because of the skewed data distribution. The preprocessed matrix was subject to pairwise comparison based on student t-test and Pearson’s correlation analysis with the correlation parameters.

**Bioinformatics analysis**

Canonical pathway enrichment was performed using Ingenuity Pathway Analysis (IPA, QIAGEN, Hilden, Germany) based on the significantly correlated protein with clinical parameters. The confidence level is reflected in the *P* value of Fisher's exact test, and the extent of activation is reflected by the Z-score. Here, the *P* value cutoff for enrichment was 0.05, and the predictive activation Z-score cut-off was unity. After protein-protein interactions of the selected proteins were obtained from the String version 11 database (https://string-db.org) [8], network models were constructed using Cytoscape version 3.10 software [9].

**Supplementary Reference**

1. Park J, Kim H, Kim SY, Kim Y, Lee JS, Dan K, et al. In-depth blood proteome profiling analysis revealed distinct functional characteristics of plasma proteins between severe and non-severe COVID-19 patients. Sci Rep. 2020;10:22418. doi:10.1038/s41598-020-80120-8

2. Kwon S, Cheon S, Kim KH, Seo A, Bae E, Lee JW, et al. Unveiling the role of transgelin as a prognostic and therapeutic target in kidney fibrosis via a proteomic approach. Exp Mol Med. 2024;56:2296-308. doi:10.1038/s12276-024-01319-7

3. Suh J, Han D, Ku JH, Kim HH, Kwak C, Jeong CW. Next-generation Proteomics-Based Discovery, Verification, and Validation of Urine Biomarkers for Bladder Cancer Diagnosis. Cancer Res Treat. 2022;54:882-93. doi:10.4143/crt.2021.642

4. Reiter L, Rinner O, Picotti P, Hüttenhain R, Beck M, Brusniak MY, et al. mProphet: automated data processing and statistical validation for large-scale SRM experiments. Nat Methods. 2011;8:430-5. doi:10.1038/nmeth.1584

5. Lou R, Cao Y, Li S, Lang X, Li Y, Zhang Y, et al. Benchmarking commonly used software suites and analysis workflows for DIA proteomics and phosphoproteomics. Nat Commun. 2023;14:94. doi:10.1038/s41467-022-35740-1

6. Welter AS, Gerwien M, Kerridge R, Alp KM, Mertins P, Selbach M. Combining Data Independent Acquisition With Spike-In SILAC (DIA-SiS) Improves Proteome Coverage and Quantification. Mol Cell Proteomics. 2024;23:100839. doi:10.1016/j.mcpro.2024.100839

7. Tyanova S, Temu T, Sinitcyn P, Carlson A, Hein MY, Geiger T, et al. The Perseus computational platform for comprehensive analysis of (prote)omics data. Nat Methods. 2016;13:731-40. doi:10.1038/nmeth.3901

8. Szklarczyk D, Franceschini A, Wyder S, Forslund K, Heller D, Huerta-Cepas J, et al. STRING v10: protein-protein interaction networks, integrated over the tree of life. Nucleic Acids Res. 2015;43:D447-52. doi:10.1093/nar/gku1003

9. Shannon P, Markiel A, Ozier O, Baliga NS, Wang JT, Ramage D, et al. Cytoscape: a software environment for integrated models of biomolecular interaction networks. Genome Res. 2003;13:2498-504. doi:10.1101/gr.1239303
